# Relationship between sheep feces scores and gastrointestinal microorganisms and their effects on growth traits and blood indicators

**DOI:** 10.3389/fmicb.2024.1348873

**Published:** 2024-02-14

**Authors:** Xiaobin Yang, Jianghui Wang, Jiangbo Cheng, Deyin Zhang, Kai Huang, Yukun Zhang, Xiaolong Li, Yuan Zhao, Liming Zhao, Dan Xu, Zongwu Ma, Jia Liu, Zhiqiang Huang, Chong Li, Huibin Tian, Xiuxiu Weng, Weimin Wang, Xiaoxue Zhang

**Affiliations:** ^1^College of Animal Science and Technology, Gansu Agricultural University, Lanzhou, Gansu, China; ^2^The State Key Laboratory of Grassland Agro-ecosystems, College of Pastoral Agriculture Science and Technology, Lanzhou University, Lanzhou, Gansu, China

**Keywords:** Hu sheep, fecal scores, digestive tract microorganisms, growth performance, blood physiology and biochemistry

## Abstract

Fecal scores are crucial for assessing the digestive and gastrointestinal status of animals. The Bristol fecal scoring system is a commonly used method for the subjective evaluation of host feces, there is limited research on fecal scoring standards for fattening Hu sheep. In this study, Hu sheep were collected for rumen, rectum, and colon contents for 16S rDNA sequencing. 514 Hu sheep feces were scored based on the Bristol fecal scoring system, and production performance at each stage was measured. Finally, we developed the scoring standard of the manure of Hu sheep in the fattening period (a total of five grades). The result shows that moisture content significantly increased with higher grades (*p* < 0.05). We analyzed the relationship between fecal scores and production traits, blood indices, muscle nutrients, and digestive tract microorganisms. The growth traits (body weight, body height, body length, average daily gain (ADG), and average daily feed intake (ADFI) during 80–180 days), body composition traits of the F3 group, and the carcass traits were found to be significantly higher (*p* < 0.05) than those of the F1 and F2 groups. There was no significant difference in gastrointestinal microflora diversity among all groups (*p* > 0.05). Significant differences were observed in Aspartate aminotransferase, Glucose, Total bilirubin, and Red Blood Cell Count between groups (*p* < 0.05). The mutton moisture content in group F4 was significantly higher than in the other groups, and the protein content was also the lowest (*p* < 0.05). The results of the correlation analysis demonstrated that Actinobacteria, *Peptostreptococcaceae*, Acidaminococcales, Gammaproteobacteria, and Proteobacteria were the significant bacteria affecting fecal scores. In addition, *Muribaculaceae* and *Oscillospiraceae* were identified as the noteworthy flora affecting growth performance and immunity. This study highlights the differences in production traits and blood indicators between fecal assessment groups and the complex relationship between intestinal microbiota and fecal characteristics in Hu sheep, suggesting potential impacts on animal performance and health, which suggest strategies for improved management.

## Introduction

Feces include feed residues, metabolic byproducts, mucus secreted by digestive glands, epithelial cells shed from gastrointestinal mucosa, and metabolic waste (Fernández-Ibáñez et al., 2009; Lyons et al., 2016). The morphology of fecal patterns can somewhat indicate the gastrointestinal health of livestock. The Bristol fecal scoring system (BSFS), proposed by Heaton et al. in 1997, divides fecal matter into 7 morphology-based grades, which are mainly used to identify changes in human intestinal function (Nordin et al., 2022). The BSFS has emerged as a standard for consistent fecal forms and is widely employed in research and medical settings. The manure-scoring system for pigs and cattle is relatively mature (Renaud et al., 2020; Wang et al., 2021), while the manure-scoring system for sheep is rarely reported. Fecal scoring provides a means of evaluating the digestive health of animals. The timely detection of an animal’s digestive problems can be achieved by evaluating and comparing the color, shape, and odor of feces, enabling the implementation of appropriate dietary adjustments and feeding management measures. Hence, fecal scoring holds significant importance in animal production and management.

Gut microflora plays a critical role in host health. It has the potential to regulate gastrointestinal physiology and pathophysiology (de Vos et al., 2022). Intestinal flora produces bioactive small molecule metabolites that act as ligands for G protein-coupled receptors and hormone receptors, such as short-chain fatty acids, lipids, N-acrylamides, and amino acid metabolites, thereby affecting animal growth and development (Tanes et al., 2021). Numerous studies have demonstrated that rumen microflora influences ruminant growth traits. In a study by Daghio et al. (2021), it was found that the relative abundance of bovine rumen microorganisms *Ruminococcaceae UCG-01*, *Treponema 2*, and *Lachnospiraceae NK3A20 group* had a negative correlation with growth performance, whereas the relative abundance of *Succinivibrionaceae UCG-002*, *Rikenellaceae RC9 gut group*, and *Fibrobacter* had a positive correlation with growth performance. The colon performs the role of absorbing water and electrolytes and regulating the expulsion of intestinal substances. The colon microflora interacts with various components of the colon, such as different types of mucosal epithelial cells, immune response cells, and neuromuscular tissue, to maintain the normal functioning of the colon (Moran and Jackson, 1992). Fecal dominant phyla include Firmicutes, Bacteroidetes, Proteobacteria, and Fusobacteria, and fecal microorganisms have also been associated with host growth and fat deposition (Chen et al., 2021; Zhao et al., 2022). Gastrointestinal microorganisms are closely related to host growth, development, and immunity.

The fecal score can effectively reflect animals’ digestive function and health status, and the BSFS is currently widely used. The mature rumen has important physiological functions such as absorption, transport, metabolic activity, and host protection, and the dysfunction of the intestinal mucosal barrier is an important pathophysiological feature of diarrhea. Fecal scores are widely used to judge the degree of diarrhea in animals, so previous studies have focused on diarrhea in young animals, but there have been few studies on changes in fecal scores in mature animals of the microflora, the relationship between fecal scores and phenotypes, and the effects of microorganisms on fecal scores. Therefore, this study conducted performance measurement and fecal morphology evaluation on a large scale of Hu sheep, aiming to establish a fecal scoring standard of Hu sheep, explore the relationship between fecal score and production performance and immune performance, and provide a reference for quickly understanding the growth and health status of Hu sheep.

## Materials and methods

### Animals and management

In this study, 514 healthy Hu sheep male lambs from Hu sheep breeding farms in Wuwei, Gansu, Hangzhou, Zhejiang, and Huzhou, Zhejiang were selected as test subjects. All lambs were weaned at 56 days of age, and after weaning, they were transferred to the experimental base in Minqin (Minqin County, Gansu Province, China, N38°34′, E102°59′, altitude 1,378 m) for single pen rearing. Pellets were fed during the all experimental period, and the experimental feeds were formulated according to the recommended formula of the Chinese sheep feeding standard (NY/T816-2004), the ration formula is shown in Supplementary Table S1, and the pellets were made by Gansu Runmu Bioengineering Limited Liability Company (Jinchang City, Gansu Province, China). All sheep underwent routine immunization procedures administered by specialized veterinarians. The test period included 14 days of the transition period, 10 days of the pre-feeding period, and 100 days of the test period, and slaughter was conducted at the end of the feeding test. Each single pen was equipped with a separate feeding trough and drinking trough, and all sheep fed and drank freely. All sheep were managed in the same way and at the same nutritional level.

### Traits measurement and sample collection

All sheep were subjected to traits measurements (body weight, body height, body length, chest circumference, and cannon circumference) before morning feeding (having fasted for more than 12 h) at 80, 100, 120, 140, 160, and 180 days of age. The feed intake of each sheep was recorded every 10 days, and the difference between the feed intake and food refusal was taken as the ADFI. The calculation of feed conversion ratio (FCR) was performed according to Horodyska et al. (2017), which is (FCR) = ADFI/ADG.

All sheep were slaughtered at 180 days of age. The visceral organs of all sheep were divided according to tissue parts stripped of fat and weighed and recorded as absolute weight. Rumen content samples were collected from each sheep in 5 mL cryotube after slaughter (*n* = 487). Based on the grouping of fecal scores as described above, 15 Hu sheep near the median of each group were selected and feces and colon contents were collected in a 5 mL cryotube, respectively. The samples were stored at −80°C. They were subsequently sent to Novogene (Beijing, China) for 16S rDNA sequencing.

Before slaughter, 2.5 mL and 5 mL blood were collected from the jugular vein of 180-day-old Hu sheep, respectively, for the determination of blood physiological and biochemical indexes. Blood physiological indexes were determined by Mindary Automatic Animal Blood Cell Analyser BC-2800 Vet (Shenzhen, China). The collected 5 mL of blood was centrifuged, and the serum was immediately transferred to a centrifuge tube and stored in an ultra-low-temperature refrigerator at −80°C, and the blood biochemical indexes were measured by Mindary automatic biochemical analyzer BS-350S (Shenzhen, China). The collected rumen fluid was measured by a PANNA gas chromatograph (A91PLUS, PANNA Instruments Co., Ltd., China); the chromatographic column was an S.N17-11-010 capillary column (Analytical Technology, China); the chromatographic conditions were inlet temperature of 250°C, nitrogen flow rate of 5.4 mL/min, shunt ratio of 5:1, injection volume of 1 μL, programmed heating mode (190°C for 3 min, then 30°C/min to 240°C, hold for 1 min), flame ionization detector (FID) 250°C, and FID air, hydrogen and nitrogen flow rates of 300, 30 and 20 mL/min, respectively. The samples of the longest back muscle of Hu sheep were collected for the determination of routine nutrient composition (fat, moisture, salt, protein, and collagen). The routine nutrient composition determination of the longest back muscle was performed based on (Foodscan 2, fosschina, China, Beijing) with three biological replicates for each sample and two technical replicates for each biological replicate.

### Fecal morphology observation and moisture determination

With reference to the BSFS, staff were first arranged to train Hu sheep manure scoring. At the age of 140, 160, and 180 days of Hu sheep, the fresh feces of Hu sheep were observed and recorded by fixed personnel (to minimize the error caused by subjective factors) and then collected for the determination of fecal moisture content according to the method specified in the national standard GB5009.3–2016. Finally, Hu sheep manure scoring criteria at the fattening stage were established according to morphology and water content and with reference to the BSFS. The formula for moisture calculation was as follows: total moisture content of feces (%) = (mass of fresh feces - mass of dried feces sample at 105°C)/mass of fresh feces × 100.

### Library construction and sequencing

The microbial DNA extraction method was referred to by Zhang et al. (2021). DNA from rumen contents was extracted using the EasyPure Stool Genomic DNA Kit (All Style Gold Biotech Co., Ltd., Beijing, China). The concentration and purity of DNA were checked on a 1% agarose gel, and the concentration of DAN was diluted to l ug/μL using sterile water. The library was constructed using the TruSeq® DNA PCR-Free Sample The libraries were constructed using the TruSeq® DNA PCR-Free Sample Preparation Kit, and the quality of the libraries was assessed by Qubit@2.0 Fluorometer (Thermo Scientific) and Agilent Bioanalyzer 2,100 platform, and the assessed libraries were subjected to high-throughput bipartite sequencing on the Illumina NovaSeq platform.

### Species annotation

After sequencing, bipartite reads for each sample were differentiated and matched based on specific sample barcodes, and the barcodes and primer sequences were cut. The FLASH program merged the bipartite reads^1^ to generate Raw tags. The quality control process used QIIME (v1.9.1, http://qiime.org/scripts/split libraries fastq.html) to filter the raw tags and generate clean tags. The UCHIME algorithm^2^ was used to compare the clean tags with a reference database (Silva database, https://www.arb-silva.de/) to remove chimeric sequences. The final result is valid tags (Clean Data) for subsequent analysis. Sequences were de-weighted (dereplication) using QIIME 2 and clustered with 100% similarity (Identity = 100%) to obtain ASVs (Amplicon Sequence Variants, ASVs) sequences. And the species annotations of ASV sequences were performed in the Silva138 database.

### Data analysis

Alpha diversity indices of samples were calculated using Qiime software (Version 1.7.0). Beta diversity was calculated using R (vegan package) to calculate sample distances (Bray-curtis) and plot PCoA. Differential microbes were analyzed using the galaxy platform.^3^ All means and standard errors were statistically derived from SPSS 26.0, and one-way ANOVA and LSD were used to test for differences between groups. Spearman correlation analysis was performed using RStudio software (openxlsx package, pheatmap package, psych package).

## Results

### Fecal scoring

**Figure 1:**
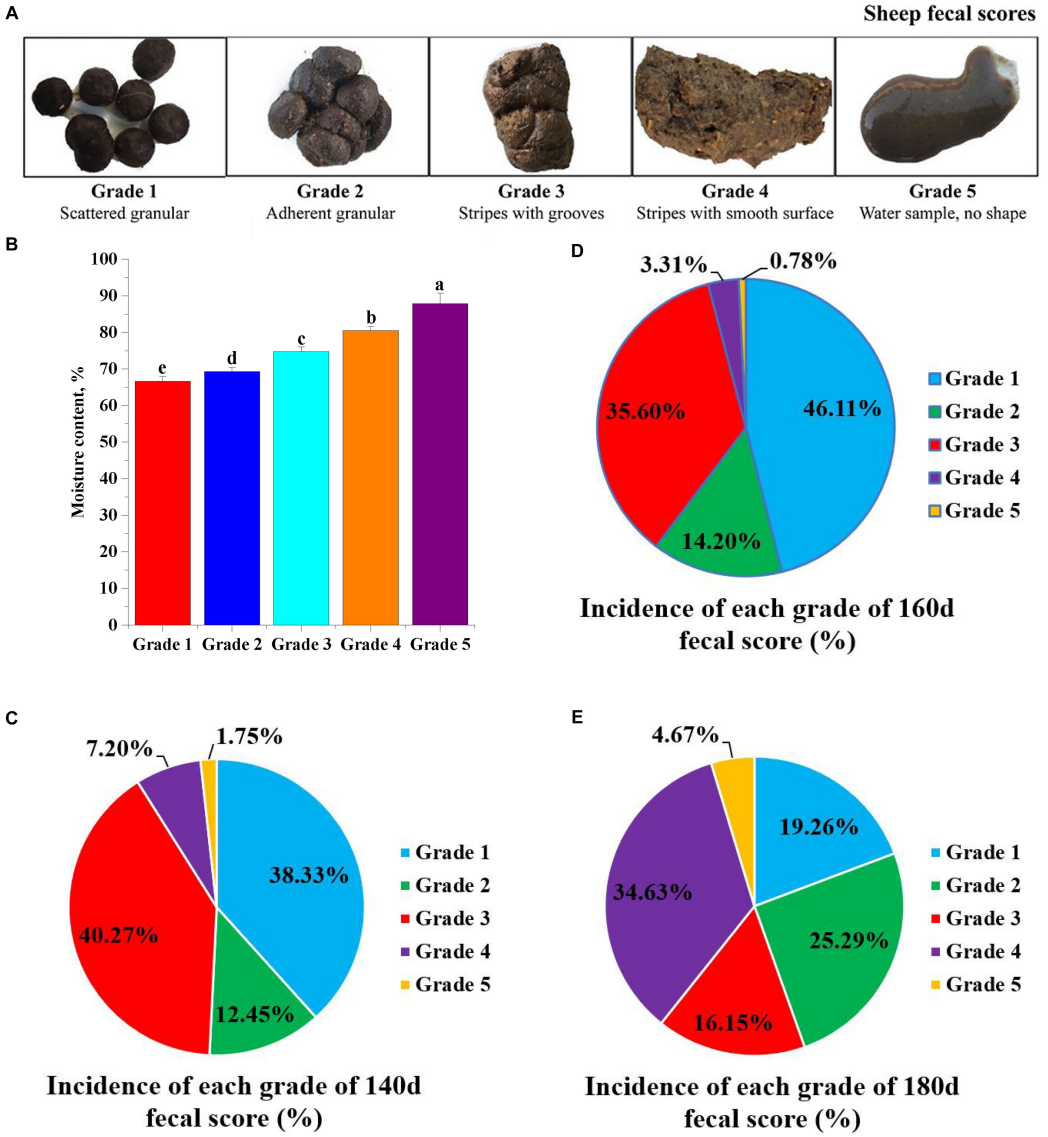
Fecal scoring of Hu sheep. **(A)** Different fecal scoring grades of fecal morphology. **(B)** The moisture content of different fecal scoring levels. Different lowercase letters indicate significant differences. **(C–E)** Incidence of different fecal score grades at 140d, 160d, and 180d.

After observing the fecal patterns of 514 sheep for 140 d, 160 d, and 180 d, we finally formulated the fecal scoring grades (five grades in total) for the fattening period of the sheep, and the results are shown in Figure 1A. Subsequently, the moisture content of the feces of different grades was measured (Figure 1B), and it was found that there were significant differences in moisture content among the grades. Grade 1 is dispersed granular manure with a moisture content of about 67%; grade 2 is intact pellets bonded together with a moisture content of about 69%; grade 3 has a long, stringy shape with a strangled surface and a moisture content of about 75%; grade 4 has a long, stringy shape with a smooth surface and a moisture content of about 81%; and grade 5 is soft, watery manure of no defined shape and a moisture content of about 88%. The moisture content of the feces increased significantly (*p* < 0.05) as the grade of fecal score increased. As shown in Figures 1C–E, the prevalence of grades 1 and 3 was higher at 140 and 160 days of age, ranging from 35 to 46%, and grade 4 was the highest at 180 days of age, with a prevalence of 34.63%.

### Characterization of rumen, colon, and rectum microflora of Hu sheep with different fecal scores

#### Sequencing data presentation

In this study, 16S rDNA sequencing was performed on rumen microbes of 487 Hu sheep, colon microbes of 36 Hu sheep, and rectal microbes of 45 Hu sheep. As shown in Supplementary Figure S1, the dilution curves of the rumen, colon, and rectal samples all leveled off, indicating that the depth could cover the vast majority of the microorganisms in each sample, which was in line with the analytical needs.

#### Diversity analysis

Based on the mean values of fecal scores of the test sheep at three stages of 140, 160, and 180 days of age, all the test sheep were divided into four groups, i.e., group F1 (1 ≤ fecal score < 2), group F2 (2 ≤ fecal score < 3), group F3 (3 ≤ fecal score < 4), and group F4 (4 ≤ fecal score < 5).

**Figure 2:**
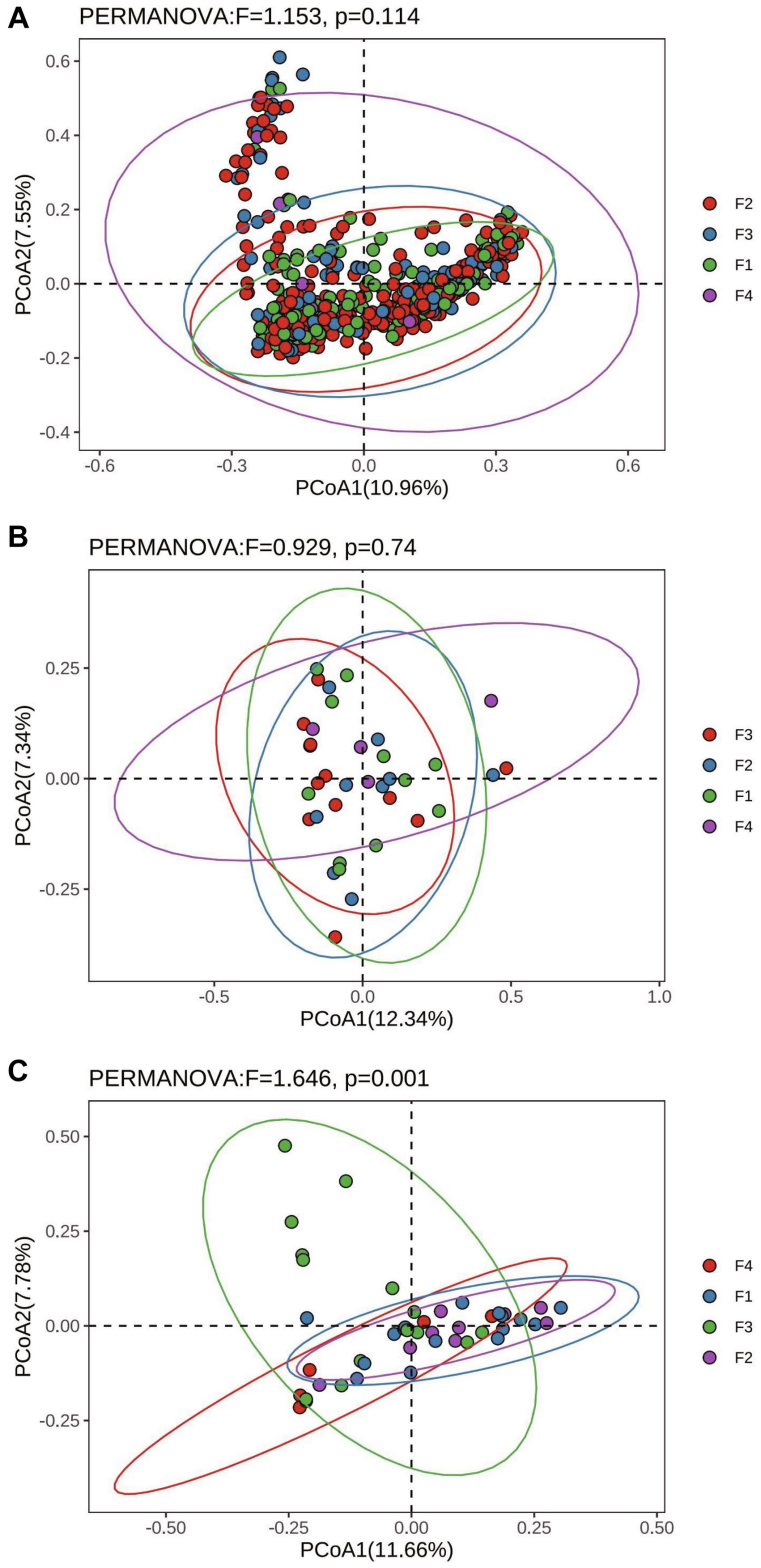
Principal Co-ordinates analysis based on Bray_curtis distances in Hu sheep with different fecal scores. **(A)** Rumen; **(B)** colon; **(C)** rectum.

The results of Alpha diversity of rumen, colon, and rectum in different fecal scoring groups are shown in Supplementary Table S2. The results showed that the Simpson index in the rumen of group F1 was significantly higher than that of group F3 (*p* < 0.05), and the Chao 1 index of group F4 was significantly higher than that of group F1 (*p* < 0.05). There was no significant difference (*p* > 0.05) between the groups of Chao 1, Shannon, and Simpson in the colon and rectum. Bray_curtis distances were calculated separately for rumen, colon and rectum based on the ASV data and the Permanova test was performed. The PCoA (Principal Coordinates Analysis) results of rumen, colon, and rectum based on the Bray_curtis distances showed overlap between the F1, F2, F3, and F4 groups in different positions (Figure 2).

#### Analysis of species composition

**Figure 3:**
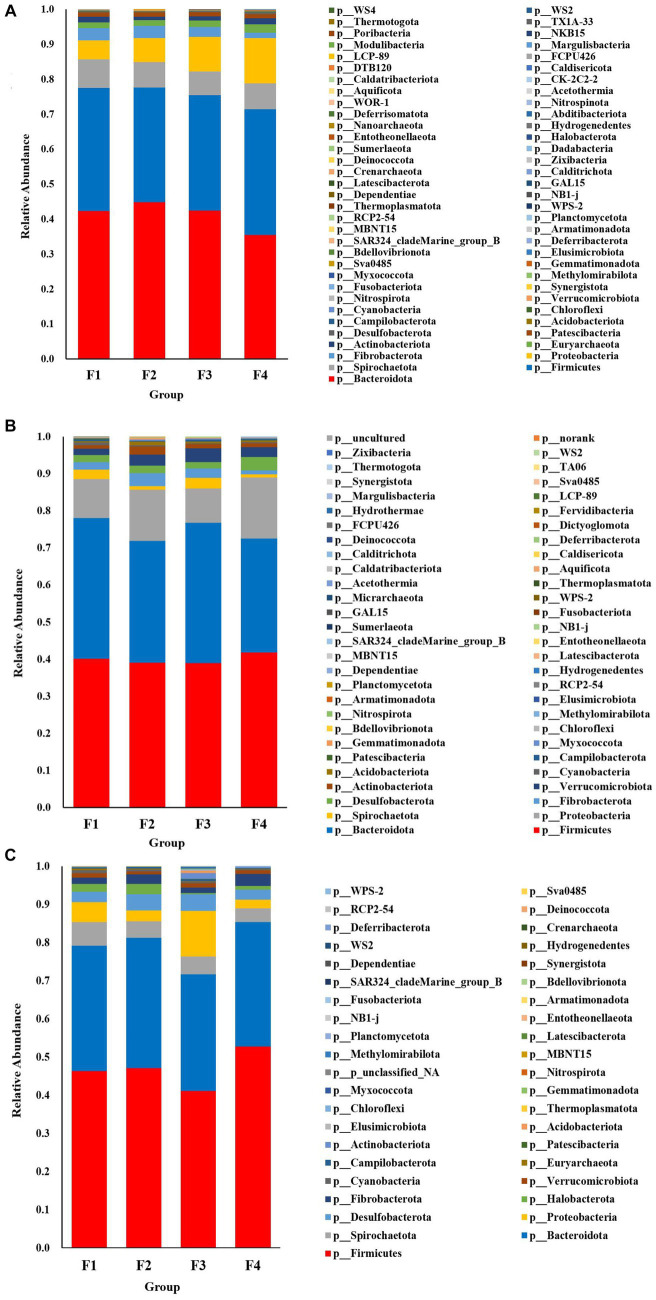
Species composition at the phylum level in Hu sheep with different fecal scores. **(A)** Rumen; **(B)** colon; **(C)** rectum.

**Figure 4:**
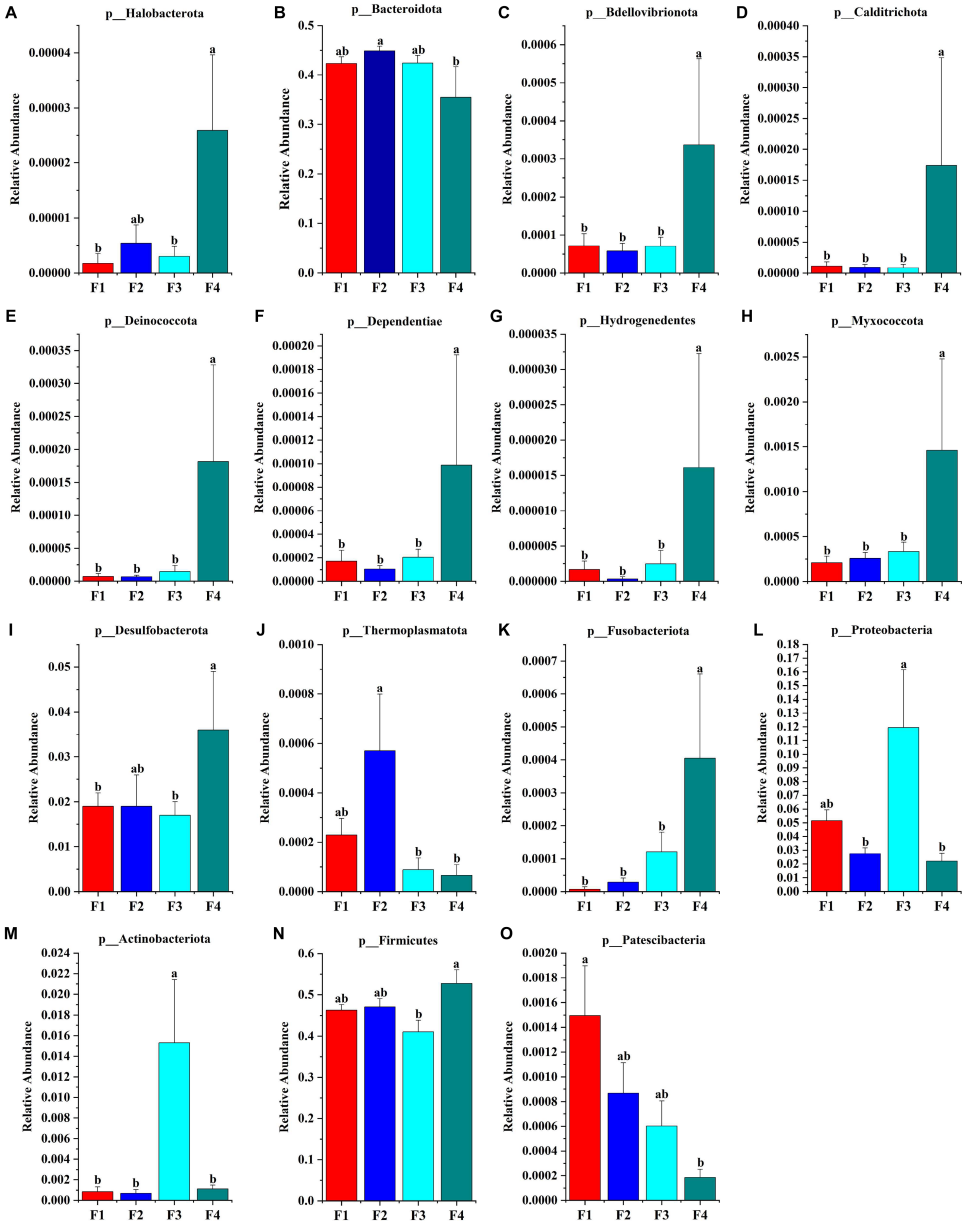
Microbial phylum that differ in Hu sheep between groups with different fecal scores. **(A–H)** Rumen; **(I)** colon; **(J–O)** rectum. Different lowercase letters indicate significant differences (*p* < 0.05).

The species accumulation diagrams for the rumen, colon, and rectal are shown in Figure 3. A total of 65 phyla were identified in the rumen. The dominant phyla in the rumen were Bacteroidota, Firmicutes, Spirochaetota, Proteobacteria, and Fibrobacterota. A total of 56 phyla were identified in the colon, and the dominant phyla in the colon were Firmicutes, Bacteroidota, Proteobacteria, Fibrobacterota, and Verrucomicrobiota. A total of 41 phyla were identified in the rectum and the dominant phyla in the rectum were Firmicutes, Bacteroidota, Spirochaetota, Desulfobacterota, and Proteobacteria. Differential microorganisms at the phylum level in the rumen, colon, and rectum are shown in Figure 4. There were eight rumen differential phyla in different fecal scoring groups of Hu sheep, namely Halobacterota, Bacteroidota, Bdellovibrionota, Calditrichota, Deinococcota, Dependentiae, Hydrogenedentes and Myxococcota (*p* < 0.05). One differential phylum of colon, Desulfobacterota (*p* < 0.05). six rectal differential phyla, Thermoplasmatota, Fusobacteriota, Proteobacteria, Actinobacteriota, Firmicutes, and Patescibacteria (*p* < 0.05). The relative abundance of Halobacterota, Bdellovibrionota, Calditrichota, Deinococcota, Dependentiae, Hydrogenedentes, and Myxococcota in the rumen was significantly higher in group F4 than in the other three groups (*p* < 0.05). The relative abundance of Desulfobacterota was significantly higher in the colon F4 group than in the F1 and F3 groups (*p* < 0.05). In the rectum, Proteobacteria and Actinobacteriota had the highest relative abundance in group F3; Fusobacteriota and Firmicutes had a significantly higher relative abundance in group F4 than in the other three groups; Patescibacteria had the highest relative abundance in group F1, and Thermoplasmatota in group F2 (*p* < 0.05).

In order to compare the differential microorganisms in the rumen, colon, and rectum of Hu sheep in different fecal scoring groups, we performed LEfSe analysis with the setting of LDA ≥ 2, and the results are shown in Supplementary Figure S2. A total of three differential microorganism were identified in the rumen, *Eubacterium_sp_AB3007* and *Pseudoxanthomonas* enriched in group F4 and *Alloprevotella_s_uncultured_organism* enriched in group F2. A total of 11 differential microorganism were identified in the colon, *Cellvibrionaceae*, *Cellvibrio*, *Ensifer_adhaerens* enriched in group F1, Actinobacteria, *Caproiciproducens*, *Caproiciproducens-s_uncultured*_bacterium, *Peptostreptococcaceae-g_uncultured-s_uncultured_bacterium*, *Peptostreptococcaceae-g_uncultured*, *Verrucomicrobia_bacterium* were enriched in group F2 and Cellvibrionales, *Cellvibrio_sp_OA_2007* were enriched in group F4. A total of 21 differential microorganism were identified in the rectum, Acidaminococcales, *Acidaminococcaceae*, Negativicutes, Gammaproteobacteria, *Succinivibrio*, *Succinivibrionaceae*, Aeromonadales, Proteobacteria enriched in group F1, *Prevotella*, Archaea, enriched in group F2, *Phascolarctobacterium*, *Desulfovibrio*, Desulfobacterota, Desulfovibrionia, Desulfovibrionales, *Desulfovibrionaceae* enriched in group F3, Peptostreptococcales_Tissierellales, *Muribaculaceae*, *Oscillospiraceae_g_uncultured* enriched in group F4.

#### Differential microbial and phenotypic correlation analysis

**Figure 5:**
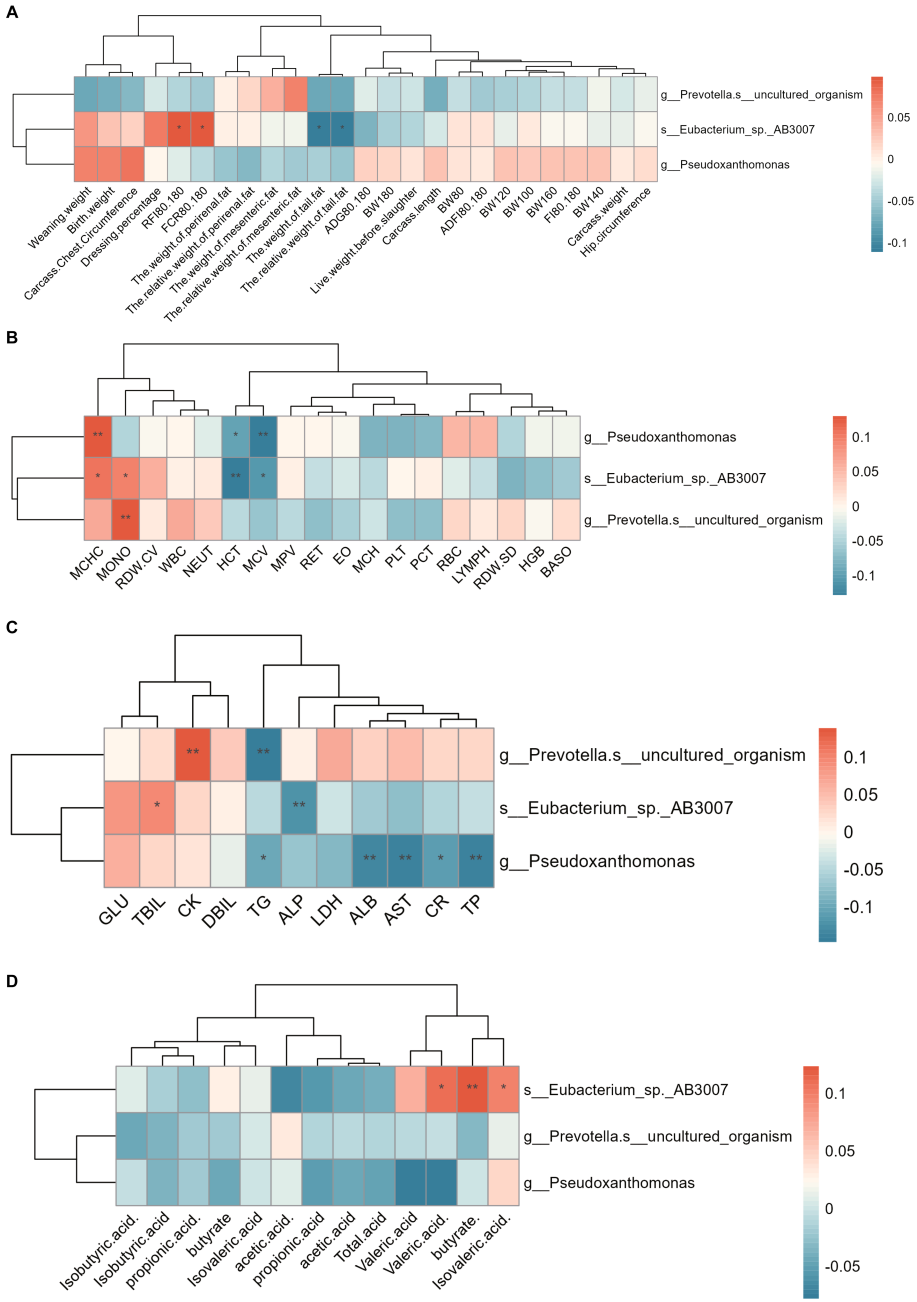
Spearman correlation analysis between rumen differential microflora and phenotype. **(A)** Correlation analysis between rumen differential microflora and production performance. **(B)** Correlation analysis between rumen differential microflora and blood physiological indexes. **(C)** Correlation analysis between rumen differential microflora and blood biochemical indices. **(D)** Correlation analysis between rumen differential microflora and VFA. ^*^ means *p* < 0.05, ^**^ or ^***^ means *p* < 0.01. The same below.

Correlation analyses were performed in order to investigate the correlation between differential microorganism in the rumen, colon, and rectum and the phenotypes of Hu sheep. The results of the correlation between rumen differential microorganism and phenotypes are shown in Figure 5. The results showed that *Eubacterium_sp_AB3007* was significantly and positively correlated with FCR and residual feed intake (RFI), Mean corpuscular Hemoglobin Concentration (MCHC), Monocyte Count (MONO), Total bilirubin (TBIL), the percentage of valeric acid, butyrate, and isovaleric acid in Hu sheep from 80–180 d, and was significantly and negatively correlated with the (relative) weight of tail fat, Hematokrit (HCT), Mean Corpuscular Volume (MCV), and Alkaline phosphatase (ALP) (*p* < 0.05). *Pseudoxanthomonas* was significantly positively correlated with MCHC and significantly negatively correlated with HCT, MCV, Triglyceride (TG), Albumin (ALB), Aspartate aminotransferase (AST), Creatinine (CR), and Total protein (TP) (*p* < 0.05). *Prevotella_s_uncultured_organism* was significantly positively correlated with MONO, and Creatine kinase (CK) and negatively associated with TG (*p* < 0.05). *Prevotella_s_uncultured_organism* was significantly positively correlated with HCT and TG (*p* < 0.05). *Pseudoxanthomonas* was significantly negatively correlated with HCT and TG and significantly negatively correlated with TG (*p* < 0.05).

**Figure 6:**
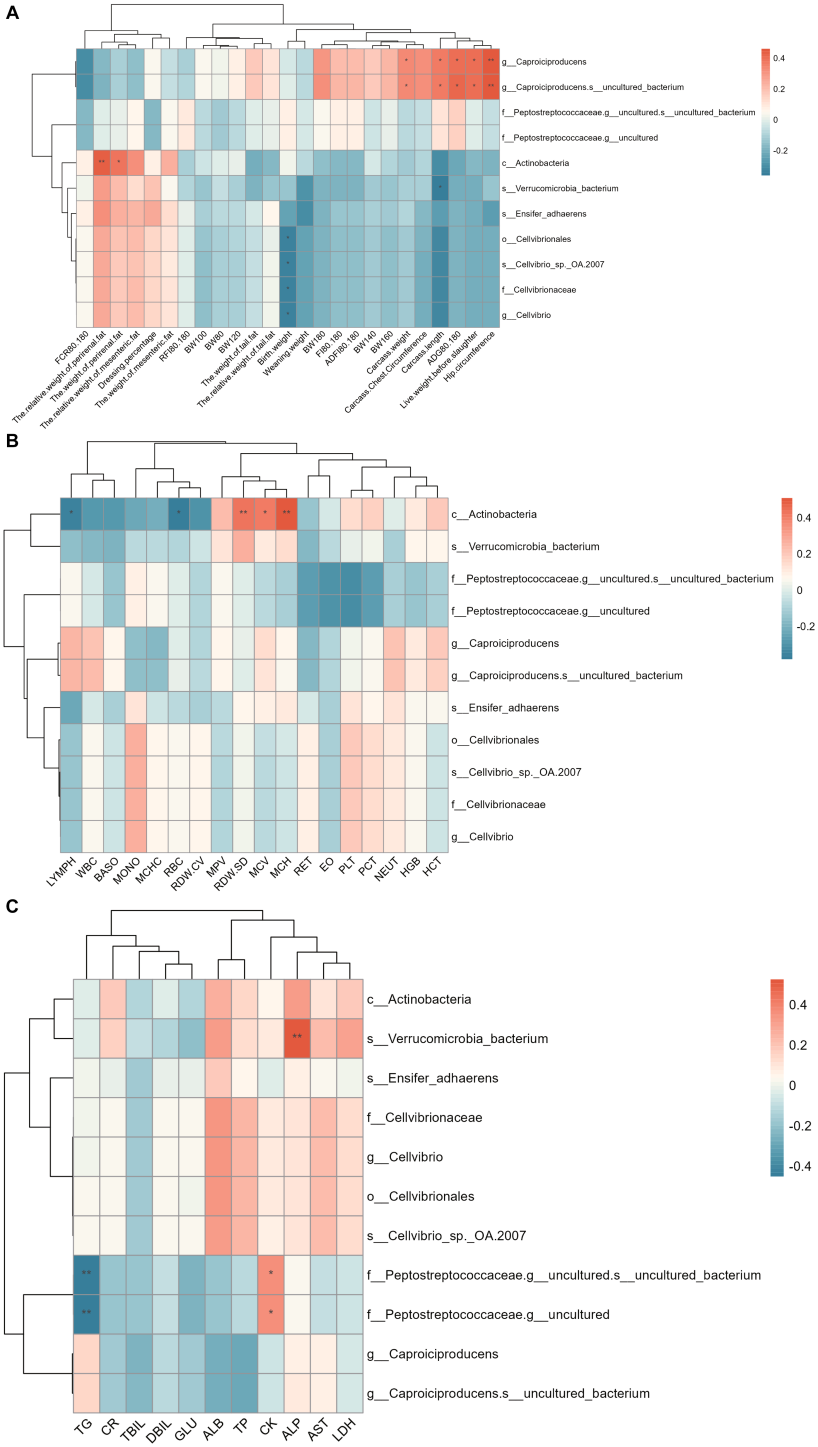
Spearman correlation analysis between colon differential microflora and phenotype. **(A)** Correlation analysis between colon differential microflora and production performance. **(B)** Correlation analysis between colon differential microflora and blood physiological indexes. **(C)** Correlation analysis between colon differential microflora and blood biochemical indices.

The correlation between colonic differential microbes and phenotypes is shown in Figure 6. The results showed that *Caproiciproducens* and *Caproiciproducens-s_uncultured_bacterium* were significantly positively correlated with pre-slaughter live weight, carcass weight, carcass length, carcass hip circumference, and ADG of 80–180 d. Actinobacteria is significantly positively correlated with perineal fat weight and relative weight, Red Cell Distribution Width-Standard Deviation (RDW-SD), MCV, and Mean Corpuscular Hemoglobin (MCH), and negatively correlated with LYPMH and Red Blood Cell Count (RBC). *Verrucomicrobia_bacterium* was significantly positively correlated with ALP and negatively correlated with carcass length. There was a significant negative correlation between Cellvibrionales and the birth weight of Hu sheep. *Peptostreptococceaceae-g_uncultured* was significantly positively correlated with CK and negatively correlated with TG.

**Figure 7:**
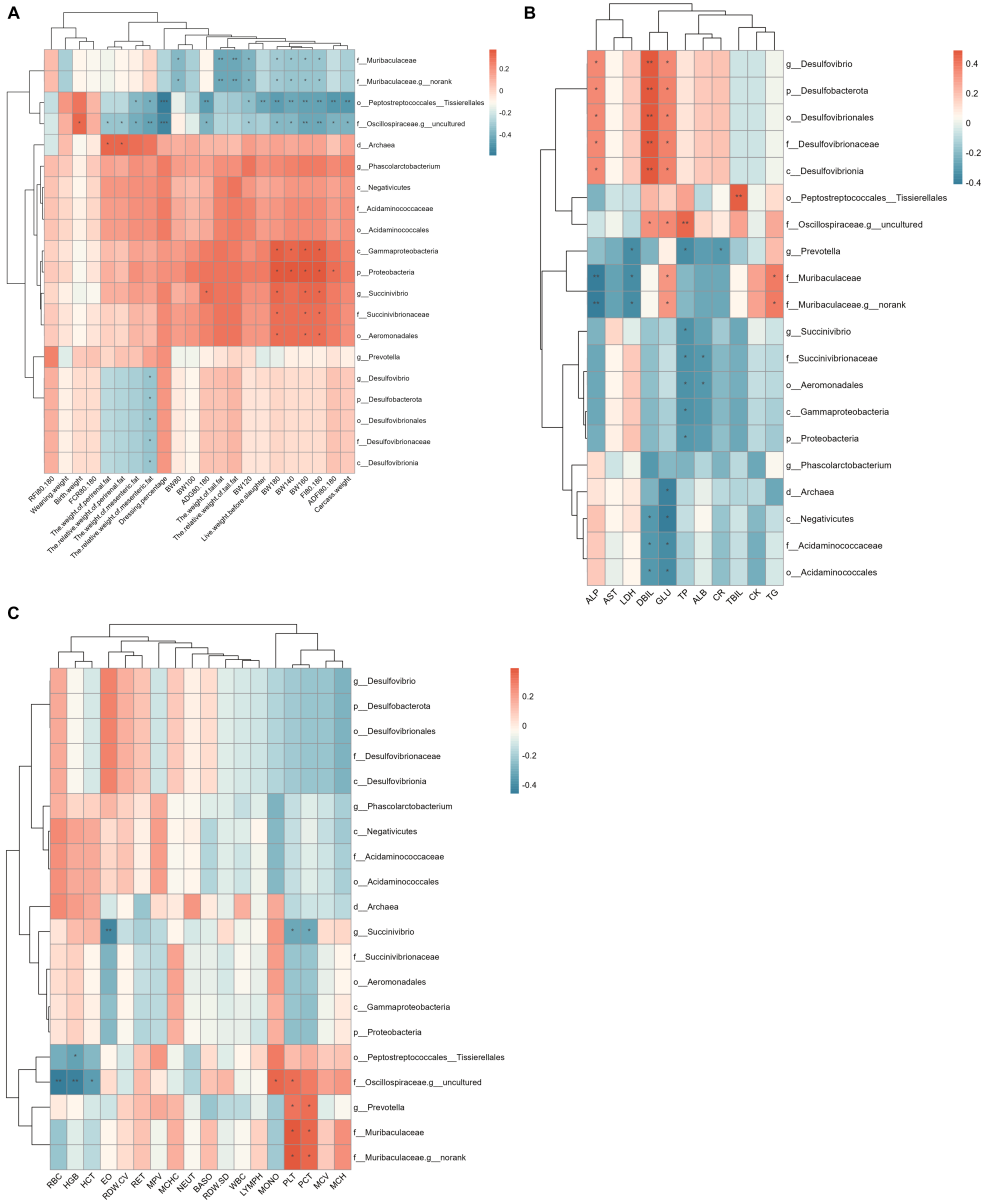
Spearman correlation analysis between rectum differential microflora and phenotype. **(A)** Correlation analysis between rectum differential microflora and production performance. **(B)** Correlation analysis between rectum differential microflora and blood physiological indexes. **(C)** Correlation analysis between rectum differential microflora and blood biochemical indices.

The correlation between rectal differential microbes and phenotype is shown in Figure 7. The results showed that Peptostreptococcales_Tissierellales, *Muribaculaceae*, and *Oscillospiraceae_g_uncultured* were significantly negatively correlated with body weight and tail fat weight of Hu sheep. *Prevotella*, *Muribaculaceae* were significantly positively correlated with Platelet (PLT) and Thrombocytocrit (PCT). Desulfobacterota is significantly and positively correlated with ALP, Direct bilirubin (DBIL), and GLU. Acidaminococcales were significantly negatively correlated with DBIL and Glucose (GLU).

### Comparison of production performance of different fecal scoring groups

#### Growth traits

The growth traits of Hu sheep in different fecal scoring groups were compared and the results are shown in Table 1. The results showed that the body weight of 140–180 d, ADG of 80–140 d, 80–160 d, and 80–180 d, and ADFI of 80–140 d, 80–160 d, and 80–180 d in group F3 were significantly higher than those of groups F1 and F2 (*p* < 0.05). The FCR of 80–120 d was significantly lower in group F3 than in the other three groups (*p* < 0.05). The FCR of group F3 showed a trend of being lower than that of the other three groups, and the overall trend of body height, body length, chest circumference, and cannon circumference was that group F3 was higher than that of groups F1, F2, and F4.

**Table 1:** Comparative analysis of growth traits of Hu sheep with different fecal scores.

Traits		Group				*p*-value
		F1	F2	F3	F4	
No.		135	250	121	8	
BW, kg	80d	20.32 ± 0.28	20.41 ± 0.20	20.86 ± 0.31	20.66 ± 0.90	0.554
	100d	25.45 ± 0.32	25.32 ± 0.26	26.37 ± 0.39	25.46 ± 1.65	0.137
	120d	30.93 ± 0.36^b^	30.65 ± 0.29^b^	32.18 ± 0.44^a^	30.91 ± 2.41^ab^	0.029
	140d	36.61 ± 0.41^ab^	36.56 ± 0.31^ab^	38.28 ± 0.49^a^	34.90 ± 2.97^b^	0.010
	160d	42.10 ± 0.45^ab^	42.05 ± 0.34^ab^	44.06 ± 0.53^a^	40.31 ± 3.46^b^	0.005
	180d	46.40 ± 0.46^ab^	46.68 ± 0.35^ab^	48.86 ± 0.57^a^	45.13 ± 3.27^b^	0.002
BH, cm	80d	53.08 ± 0.22	53.26 ± 0.15	53.61 ± 0.25	54.00 ± 0.68	0.324
	100d	56.63 ± 0.21^b^	56.78 ± 0.16^b^	57.52 ± 0.25^b^	59.50 ± 1.15^a^	0.001
	120d	60.01 ± 0.19^b^	60.03 ± 0.16^b^	60.69 ± 0.27^ab^	61.75 ± 1.05^a^	0.028
	140d	62.26 ± 0.20^b^	62.33 ± 0.16^b^	63.00 ± 0.24^ab^	63.50 ± 0.98^a^	0.037
	160d	64.86 ± 0.18	64.61 ± 0.15	65.36 ± 0.27	65.25 ± 1.13	0.053
	180d	66.02 ± 0.20^b^	66.34 ± 0.15^b^	66.60 ± 0.26^b^	68.00 ± 1.56^a^	0.078
BL, cm	80d	56.82 ± 0.27	56.94 ± 0.19	57.36 ± 0.28	57.87 ± 1.06	0.395
	100d	61.30 ± 0.24^b^	61.36 ± 0.19^b^	62.41 ± 0.27^ab^	63.25 ± 1.46^a^	0.003
	120d	65.34 ± 0.23	65.19 ± 0.19	66.17 ± 0.32	65.63 ± 1.67	0.039
	140d	69.01 ± 0.24	68.67 ± 0.18	69.58 ± 0.29	69.50 ± 2.07	0.051
	160d	72.51 ± 0.24	71.89 ± 0.18	73.17 ± 0.29	71.88 ± 1.85	0.002
	180d	74.35 ± 0.24^ab^	74.21 ± 0.19^ab^	75.31 ± 0.31^a^	73.38 ± 2.06^b^	0.009
ChC, cm	80d	60.21 ± 0.29	60.46 ± 0.21	60.50 ± 0.34	61.19 ± 0.82	0.802
	100d	65.55 ± 0.29	65.37 ± 0.23	65.96 ± 0.34	64.81 ± 1.19	0.461
	120d	69.79 ± 0.29	69.62 ± 0.22	70.47 ± 0.34	69.56 ± 1.13	0.183
	140d	74.68 ± 0.30	74.48 ± 0.24	75.19 ± 0.36	72.44 ± 1.56	0.124
	160d	78.79 ± 0.29	78.94 ± 0.24	79.67 ± 0.36	76.44 ± 1.62	0.051
	180d	82.77 ± 0.32	82.71 ± 0.25	83.81 ± 0.38	82.63 ± 1.68	0.074
CaC, cm	80d	6.57 ± 0.04	6.52 ± 0.05	6.61 ± 0.07	6.78 ± 0.14	0.566
	100d	7.13 ± 0.04	7.04 ± 0.05	7.08 ± 0.07	7.35 ± 0.18	0.447
	120d	7.66 ± 0.04	7.63 ± 0.04	7.77 ± 0.04	7.53 ± 0.15	0.148
	140d	7.97 ± 0.04	7.97 ± 0.03	8.02 ± 0.08	8.03 ± 0.16	0.830
	160d	8.23 ± 0.04	8.24 ± 0.03	8.36 ± 0.040	8.33 ± 0.18	0.062
	180d	8.39 ± 0.04	8.43 ± 0.03	8.58 ± 0.05	8.56 ± 0.13	0.006
ADG, kg/d	80-100d	0.25 ± 0.01	0.24 ± 0.00	0.27 ± 0.01	0.24 ± 0.05	0.016
	80-120d	0.26 ± 0.00	0.25 ± 0.00	0.28 ± 0.01	0.25 ± 0.05	0.001
	80-140d	0.27 ± 0.00^ab^	0.26 ± 0.00^ab^	0.29 ± 0.00^a^	0.23 ± 0.04^b^	0.001
	80-160d	0.27 ± 0.00^ab^	0.27 ± 0.00^ab^	0.29 ± 0.00^a^	0.24 ± 0.04^b^	0.001
	80-180d	0.26 ± 0.00^ab^	0.26 ± 0.00^ab^	0.28 ± 0.00^a^	0.24 ± 0.03^b^	0.000
ADFI, kg/d	80-100d	1.24 ± 0.02	1.23 ± 0.02	1.28 ± 0.03	1.21 ± 0.14	0.387
	80-120d	1.41 ± 0.02	1.39 ± 0.02	1.47 ± 0.03	1.36 ± 0.16	0.066
	80-140d	1.56 ± 0.02^a^	1.55 ± 0.02^ab^	1.63 ± 0.02^a^	1.46 ± 0.17^b^	0.034
	80-160d	1.68 ± 0.02^a^	1.69 ± 0.02^ab^	1.76 ± 0.02^a^	1.57 ± 0.18^b^	0.028
	80-180d	1.77 ± 0.02^ab^	1.78 ± 0.02^ab^	1.86 ± 0.02^a^	1.68 ± 0.17^b^	0.016
FCR	80-100d	5.34 ± 0.22	5.36 ± 0.61	5.52 ± 0.61	8.79 ± 3.13	0.659
	80-120d	5.43 ± 0.08^b^	5.64 ± 0.09^b^	5.32 ± 0.09^b^	6.52 ± 1.26^a^	0.018
	80-140d	5.83 ± 0.08^b^	5.86 ± 0.06^b^	5.68 ± 0.07^b^	6.79 ± 0.66^a^	0.005
	80-160d	6.26 ± 0.07	6.29 ± 0.05	6.12 ± 0.07	6.71 ± 0.36	0.086
	80-180d	6.84 ± 0.07	6.84 ± 0.05	6.68 ± 0.07	6.92 ± 0.14	0.254

Data in the table are mean ± standard error, different lowercase letters in the same row of shoulder labels indicate significant differences, the same below. BW, Body weight; BH, Body height; BL, Body length; ChC, Chest circumference; CaC, Cannon circumference.

#### Carcass traits

Comparative analysis of carcass traits of Hu sheep with different fecal scores is shown in Table 2. Carcass chest circumference, and hip circumference of sheep in group F3 were significantly higher than those in groups F1 and F2 (*p* < 0.05). the relative weight of mesenteric fat in group F4 was significantly lower than that in the other three groups (*p* < 0.05), and group F3 was significantly lower than groups F1 and F2 (*p* < 0.05).

**Table 2:** Comparative analysis of carcass traits of Hu sheep with different fecal scores.

Traits		Group				*p*-value
		F1	F2	F3	F4	
No.		129	240	113	8	
Perirenal fat	Absolute weight, kg	0.64 ± 0.03	0.68 ± 0.03	0.59 ± 0.03	0.49 ± 0.11	0.250
	Relative weight, %	0.01 ± 0.00	0.01 ± 0.00	0.01 ± 0.00	0.01 ± 0.00	0.105
Mesenteric fat	Absolute weight, kg	0.01 ± 0.00	0.01 ± 0.00	0.01 ± 0.00	0.01 ± 0.00	0.105
	Relative weight, %	0.02 ± 0.00^a^	0.02 ± 0.00^a^	0.02 ± 0.00^b^	0.01 ± 0.00^c^	0.002
Tail fat	Absolute weight, kg	1.17 ± 0.04	1.10 ± 0.02	1.19 ± 0.04	1.09 ± 0.26	0.245
	Relative weight, %	0.03 ± 0.00	0.02 ± 0.00	0.02 ± 0.00	0.02 ± 0.01	0.266
Dressing percentage, %		0.54 ± 0.01	0.54 ± 0.00	0.54 ± 0.01	0.52 ± 0.01	0.591
Carcass weight, kg		25.23 ± 0.34	25.51 ± 0.25	26.39 ± 0.42	23.96 ± 1.83	0.074
Carcass length, cm		81.39 ± 0.35	81.01 ± 0.26	82.27 ± 0.46	82.63 ± 2.49	0.060
Carcass chest circumference, cm		74.27 ± 0.28^b^	74.70 ± 0.21^ab^	75.65 ± 0.35^a^	74.13 ± 1.48^b^	0.012
Hip circumference, cm		60.54 ± 0.24^b^	61.10 ± 0.19^ab^	61.81 ± 0.32^a^	60.63 ± 1.72^b^	0.016
Leg bone perimeter, cm		6.21 ± 0.03^b^	6.21 ± 0.04^b^	6.37 ± 0.034^ab^	6.40 ± 0.09^a^	0.039

#### Body composition traits

A comparison of the composition of the digestive tract of Hu sheep with different fecal scores are shown in Table 3. The absolute weight of rumen, absolute weight of reticulum, absolute weight of rumen, total weight of stomach, absolute weight of cecum, and absolute weight of colon were significantly higher in group F3 than in group F1 and F2 (*p* < 0.05). Duodenum weight, jejunum weight, total intestinal weight, and total weight of the digestive tract were significantly higher in group F4 than in the other three groups (*p* < 0.05).

**Table 3:** Comparative analysis of composition of the digestive tract of Hu sheep with different fecal scores.

Traits		Group				*p*-value
		F1	F2	F3	F4	
No.		119	225	98	8	
Rumen	Absolute weight, g	746.24 ± 10.66^b^	757.99 ± 8.15^b^	792.88 ± 12.02^ab^	802.13 ± 44.22^a^	0.023
	Relative weight, %	0.012 ± 0.00	0.02 ± 0.00	0.02 ± 0.00	0.02 ± 0.00	0.195
Reticulum	Absolute weight, g	115.18 ± 1.74^b^	115.63 ± 1.27^b^	121.62 ± 1.94^a^	130.99 ± 9.36^a^	0.007
	Relative weight,%	0.00 ± 0.00^b^	0.00 ± 0.00^b^	0.00 ± 0.00^b^	0.00 ± 0.00^a^	0.016
Omasum	Absolute weight, g	141.54 ± 2.60	139.62 ± 1.76	143.08 ± 2.68	138.39 ± 10.83	0.735
	Relative weight, %	0.00 ± 0.00	0.00 ± 0.00	0.00 ± 0.00	0.00 ± 0.00	0.549
Abomasum	Absolute weight, g	173.21 ± 2.97^b^	174.62 ± 2.26^b^	184.66 ± 3.38^ab^	189.34 ± 9.96^a^	0.032
	Relative weight, %	0.00 ± 0.00	0.00 ± 0.00	0.00 ± 0.00	0.00 ± 0.00	0.169
Total weight of stomach	Absolute weight, g	1176.17 ± 14.22^b^	1187.86 ± 10.59^b^	1242.23 ± 15.80^ab^	1260.85 ± 51.97^a^	0.007
	Relative weight, %	0.03 ± 0.00	0.03 ± 0.00	0.03 ± 0.00	0.03 ± 0.00	0.061
Duodenum	Absolute weight, g	37.69 ± 0.60^c^	38.63 ± 0.52^bc^	40.03 ± 0.82^b^	47.51 ± 2.08^a^	0.001
	Relative weight, %	0.000 ± 0.00^b^	0.00 ± 0.00^b^	0.00 ± 0.00^b^	0.00 ± 0.00^a^	0.001
Jejunum	Absolute weight, g	811.08 ± 12.47^d^	852.14 ± 9.15^c^	898.23 ± 13.81^b^	997.22 ± 45.18^a^	0.000
	Relative weight, %	0.02 ± 0.00^c^	0.02 ± 0.00^b^	0.02 ± 0.00^b^	0.02 ± 0.00^a^	0.000
Ileum	Absolute weight, g	27.32 ± 0.59	26.08 ± 0.41	27.12 ± 0.64	28.66 ± 3.49	0.214
	Relative weight, %	0.00 ± 0.00	0.00 ± 0.00	0.00 ± 0.00	0.00 ± 0.00	0.108
Cecum	Absolute weight, g	58.07 ± 1.05^b^	59.27 ± 0.85^b^	62.82 ± 1.24^ab^	66.09 ± 6.92^a^	0.016
	Relative weight, %	0.00 ± 0.00	0.00 ± 0.00	0.00 ± 0.00	0.00 ± 0.00	0.756
Colon	Absolute weight, g	440.04 ± 5.74^c^	457.84 ± 5.24^b^	477.15 ± 7.54^abc^	479.71 ± 33.49^a^	0.003
	Relative weight, %	0.01 ± 0.00	0.01 ± 0.00	0.01 ± 0.00	0.010 ± 0.00	0.668
Total weight of intestinal	Absolute weight, g	1368.61 ± 17.89^c^	1433.96 ± 13.09^c^	1505.35 ± 19.15^b^	1619.19 ± 57.66^a^	0.000
	Relative weight, %	0.03 ± 0.00^c^	0.03 ± 0.00^b^	0.03 ± 0.00^b^	0.04 ± 0.00^a^	0.000
Total weight of digestive tract	Absolute weight, g	2535.33 ± 31.39^c^	2621.82 ± 19.96^b^	2747.58 ± 29.99^a^	2880.05 ± 80.67^a^	0.000
	Relative weight, %	0.06 ± 0.00^c^	0.06 ± 0.00b^c^	0.06 ± 0.00^b^	0.07 ± 0.004^a^	0.000

The results of the comparison of the weight of tissue organ in different fecal scores groups are shown in Supplementary Table S3. Hoof weight, absolute weight of fur, absolute weight of liver, absolute weight of lung, and absolute weight of kidney of Hu sheep in group F3 were significantly higher than those in groups F1 and F2 (*p* < 0.05).

#### Comparison of blood indicators

The comparative analysis of blood biochemical and physiological indices of Hu sheep with different fecal scores is shown in Tables 4, 5, respectively. The level of AST was significantly higher in group F2 than in group F3 (*p* < 0.05), the level of serum GLU was significantly higher in groups F2 and F3 than in group F1 (*p* < 0.05), and the level of TBIL was significantly higher in group F4 than in the other three groups (*p* < 0.05).

**Table 4:** Comparative analysis of serum biochemical indices in Hu sheep with different fecal scores.

Traits	Group				*p*-value
	F1	F2	F3	F4	
No.	129	243	119	7	
Albumin (ALB), g/L	19.76 ± 0.52	19.97 ± 0.36	18.48 ± 0.51	19.06 ± 2.04	0.125
Alkaline phosphatase (ALP), U/L	201.64 ± 7.82	209.49 ± 5.82	186.71 ± 6.54	200.00 ± 33.31	0.133
Aspartate aminotransferase (AST), U/L	67.46 ± 2.46^ab^	71.21 ± 1.65^a^	63.06 ± 2.09^b^	66.79 ± 8.96^ab^	0.042
Creatine kinase (CK), U/L	197.37 ± 8.73	196.57 ± 6.81	173.71 ± 8.80	163.7 ± 19.84	0.159
Creatinine (CR), μmol/L	29.75 ± 0.79	29.33 ± 0.51	28.38 ± 0.74	29.3 ± 1.99	0.615
Direct bilirubin (DBIL), μmol/L	0.73 ± 0.04	0.79 ± 0.03	0.71 ± 0.04	0.85 ± 0.09	0.346
Glucose (GLU), mmol/l	7.67 ± 0.21^b^	8.37 ± 0.13^a^	8.39 ± 0.16^a^	7.83 ± 1.00^ab^	0.009
Lactate dehydrogenase (LDH), U/L	375.81 ± 11.35	388.81 ± 8.05	358.1 ± 11.05	337.86 ± 40.04	0.135
Total bilirubin (TBIL), μmol/L	0.41 ± 0.03^bc^	0.36 ± 0.02^c^	0.44 ± 0.03^b^	0.75 ± 0.17^a^	0.005
Triglyceride (TG), mmol/L	0.12 ± 0.00	0.12 ± 0.00	0.11 ± 0.00	0.13 ± 0.01	0.436
Total protein (TP), g/L	50.33 ± 1.44	50.85 ± 1.01	47.47 ± 1.36	58.64 ± 7.44	0.115

**Table 5:** Comparative analysis of blood physiological indices in Hu sheep with different fecal scores.

Traits	Group				*p*-value
	F1	F2	F3	F4	
No.	135	250	121	8	
Red Blood Cell Count (RBC), M/uL	14.25 ± 0.10^a^	14.25 ± 0.09^a^	13.82 ± 0.12^b^	13.34 ± 0.86^b^	0.009
Hemoglobin (HGB), g/dL	12.04 ± 0.06	12.03 ± 0.07	11.83 ± 0.07	11.71 ± 0.60	0.209
Hematokrit (HCT), %	33.22 ± 0.28	33.56 ± 0.25	32.57 ± 0.30	33.53 ± 1.90	0.117
Mean Corpuscular Volume (MCV), fL	23.45 ± 0.24	23.68 ± 0.18	23.74 ± 0.27	25.46 ± 1.38	0.255
Mean Corpuscular Hemoglobin (MCH), pg	8.48 ± 0.05	8.5 ± 0.05	8.61 ± 0.06	8.86 ± 0.31	0.237
Mean corpuscular Hemoglobin Concentration (MCHC), g/dL	36.45 ± 0.23	36.18 ± 0.22	36.54 ± 0.24	35.08 ± 0.85	0.455
Red Cell Distribution Width-Standard Deviation (RDW-SD), fL	26.21 ± 0.29	26.62 ± 0.27	27.19 ± 0.29	29.06 ± 1.52	0.062
Red blood cell Distribution Width-Coefficient of Variation (RDW-CV), %	42.91 ± 0.22	42.99 ± 0.18	42.27 ± 0.25	42.36 ± 0.85	0.099
Reticulocyte (RET), K/uL	3.33 ± 0.19	3.56 ± 0.14	3.49 ± 0.19	3.36 ± 0.68	0.785
Reticulocyte Percentage (RET), %	0.02 ± 0.00	0.03 ± 0.00	0.03 ± 0.00	0.03 ± 0.01	0.631
Platelet (PLT), K/uL	596.4 ± 10.78	608.05 ± 8.37	607.21 ± 13.14	632.63 ± 47.07	0.786
Mean Platelet Volume (MPV), fL	8.11 ± 0.04	8.07 ± 0.03	8.14 ± 0.05	8.01 ± 0.20	0.56
Thrombocytocrit (PCT), %	0.49 ± 0.01	0.49 ± 0.01	0.50 ± 0.01	0.51 ± 0.04	0.874
White Blood Cell Count (WBC), K/uL	12.36 ± 0.22	12.64 ± 0.19	12.79 ± 0.21	12.97 ± 1.37	0.603
Neutrophil Count (NEUT), K/uL	5.17 ± 0.15	5.27 ± 0.15	5.26 ± 0.13	5.68 ± 1.43	0.907
Lymphocyte Count (LYMPH), K/uL	5.43 ± 0.12	5.52 ± 0.08	5.56 ± 0.12	5.23 ± 0.47	0.797
Monocyte Count (MONO), K/uL	1.54 ± 0.05	1.63 ± 0.04	1.72 ± 0.06	1.81 ± 0.21	0.124
Eosinophil Count (EO), K/uL	0.12 ± 0.01	0.12 ± 0.01	0.15 ± 0.01	0.14 ± 0.03	0.132
Basophil Count (BASO), K/uL	0.09 ± 0.00	0.10 ± 0.00	0.10 ± 0.00	0.11 ± 0.02	0.444
Neutrophil Percentage (NEUT), %	41.45 ± 0.68	40.66 ± 0.62	40.98 ± 0.68	40.85 ± 5.04	0.878
Lymphocyte Percentage (LYMPH), %	44.14 ± 0.64	44.18 ± 0.57	43.59 ± 0.66	42.58 ± 3.83	0.875
Monocyte Percentage (MONO), %	12.68 ± 0.38	12.96 ± 0.28	13.48 ± 0.41	14.38 ± 1.46	0.409
Eosinophil Percentage (EO), %	0.98 ± 0.06	1.00 ± 0.04	1.15 ± 0.09	1.28 ± 0.28	0.195
Basophil Percentage (BASO), %	0.75 ± 0.03	0.80 ± 0.02	0.80 ± 0.04	0.93 ± 0.18	0.497
Reticulocyte Hemoglobin Content (RET-He), pg	13.88 ± 0.59	13.86 ± 0.33	14.38 ± 0.50	14.04 ± 2.32	0.865
Red Cell Hemoglobin Content (RBC-He), pg	10.97 ± 0.04	11.00 ± 0.03	11.01 ± 0.04	11.29 ± 0.22	0.247

### Rumen volatile fatty acids and comparison of mutton quality

The results of rumen VFA of Hu sheep in different fecal scoring groups are shown in Supplementary Table S4. The results showed that group F4 had the highest percentage of isovaleric acid and isovaleric acid, and the rest of the VFA indexes were not significantly different among the groups. In order to investigate whether there was any difference in the quality of longest back muscle of Hu sheep in different fecal scoring groups, we measured the quality of the longest back muscle, and the results are shown in Supplementary Table S5. The results showed that the moisture content of the F4 group was significantly higher than the other three groups, and the protein and collagen content of the F4 group was significantly lower than the other three groups (*p* < 0.05).

## Discussion

Sheep farming has been intensified and housed with the development of farming technology, but problems such as high farming density and low exercise are prone to the occurrence and spread of diseases. The state of feces is one of the clinical manifestations of many diseases, so appropriate fecal scoring can provide a reference for the initial assessment of the health status of Hu sheep (Xin et al., 2021). The fecal form is closely related to the moisture content of feces (Graham et al., 2018), but the moisture content of different forms of feces has rarely been reported. Therefore, in this study, we established a fecal scoring standard for Hu sheep during the fattening period based on the Bristol fecal scoring (Nordin et al., 2022) and moisture content, and the moisture content of different grades of feces differed significantly. The fecal scores at different growth stages show dynamic changes, so effective feeding management in this situation can further improve sheep performance.

The growth trait is one of the most important traits in meat livestock. It is a complex quantitative trait with medium to high heritability, and the growth performance of meat livestock is closely related to the economic benefits of farm (Van De Stroet et al., 2016). Studies on growth performance found that there were differences in the growth performance of Hu sheep in different fecal scoring groups, with body weight, ADG, body height, body length, chest circumference, and cannon circumference being higher in the F3 group than in the other three groups, which suggests that fecal scoring may reflect the growth performance of Hu sheep to a certain extent. The increase in mass and volume of visceral tissues and organs as well as the change in internal structure are important factors for the healthy growth and development of animals, which are of great significance in guiding theoretical research and production practice (Wang et al., 2021). The weight of visceral tissue organs of sheep in F3 group was significantly higher than that of F1 and F2 groups. The gastrointestinal tract of sheep is the organ and tissue of the organism that ingests food, absorbs nutrients, maintains the balance of body fluids and electrolytes, maintains the health of the organism, and discharges wastes (Cheng et al., 2010; Chase, 2018). Therefore, we speculate that sheep in group F3 may have stronger digestive absorption and immune ability. Carcass traits are important evaluation indexes for the meat production performance of livestock, some studies found that carcass traits were affected by genetic factors, sex, season, and age (Utrera and Van Vleck, 2004; Niu et al., 2021). Carcass chest circumference, carcass hip circumference, and leg bone circumference of sheep in the F3 group were significantly higher than those of the F1 and F2 groups, while carcass weight showed a significant trend. In summary, the Hu sheep in group F3 showed better production performance compared to the other groups.

Blood has multiple functions, such as the transport of gases, nutrients, hormones, anti-infective as well as coagulation roles, and hematological parameters have been correlated with immune function (Cohn, 2015). AST is an organ non-specific enzyme that regulates the metabolic activity of the body and is involved in gluconeogenesis in the liver and kidneys, glycerol *de novo* in the adipose tissue as well as in protein synthesis (Otto-Ślusarczyk et al., 2016). GLU also reflects the catabolic and anabolic capacity of the body to break down glucose and synthesize it. GLU is a major factor in the metabolism of glucose, and obesity is accompanied by insulin resistance. Metabolism and obesity are accompanied by insulin resistance, so obese animals tend to be accompanied by lower glucose catabolism (Ahmed et al., 2021). TBIL has multiple functions, signaling, regulating metabolism, antioxidants, etc., but more recent studies have found that TBIL has immunomodulatory properties and can inhibit inflammation (Jangi et al., 2013). In this study, AST was significantly higher in group F2 than in group F3, which indicated that sheep in group F2 had higher glycogen synthesis, fat degradation, and protein synthesis. GLU was significantly higher in groups F2 and F3 than in group F1, which indicated that group F2 might have higher glycogen anabolism and group F3 might have lower glucose catabolism. Erythrocytes are the most abundant blood cell type in the blood and function in gas transport and immunity (Wang et al., 2022). The mechanism of accelerated biological erythrocyte removal is immune-mediated (Mock et al., 2022), which may contribute to the lower erythrocyte content in the F3 and F4 groups.

The ecosystem composed of ruminant rumen, colonic and rectal microorganism determines the digestive and absorptive potential of ruminants and affects the productivity of the animal, so it is important to understand the composition and function of the microorganisms of the gastrointestinal tract. In this study, the Simpson’s index of the rumen of group F1 was significantly higher than that of group F3, indicating that the diversity of the rumen microbial community was significantly higher in group F3 than in group F1. The Chao1 index of the rumen of group F4 was significantly higher than that of group F1, indicating that the abundance of the rumen microbial community was significantly higher in group F4 than in group F1. The results of Beta diversity showed that the microbial composition of the rumen, colon, and rectum in different fecal score groups showed that the higher the fecal score level, the greater the difference in microbial composition among samples. The above results indicated that the microbial community composition in the rumen, colon, and rectum of Hu sheep from different fecal scoring groups was different, and the higher the fecal scoring grade, the more microbial diversity and richness increased, the results of the present study are similar to those of previous studies (Yang et al., 2019).

Bacteroidota, Firmicutes, Proteobacteria, Actinobacteria, and Spirochaetota are the dominant phyla of rumen microorganism, which agrees with the results of a previous study (Li et al., 2022). The primary function of Bacteroidota is to degrade non-fibrous substances, participate in energy metabolism, and correlate with the body’s obesity level (Ma et al., 2022). Calditrichota is able to reduce nitrate to nitrite (Youssef et al., 2019). Deinococcota is known for its ability to consume toxic substances (Battista et al., 1999). Myxococcota has been associated with fermentation, allochthonous nitrite reduction, and allochthonous sulfate reduction (Langwig et al., 2022). Desulfobacterota increased abundance related to intestinal inflammation, and it has been shown that this phylum is able to convert choline into trimethylamine, which is relevant to the health of the body (Ramireddy et al., 2021; Wang et al., 2022). Fusobacteriota is common in colorectal cancer and it is associated with an inflammatory response (Castellarin et al., 2012). Thus, different flora abundances have a role to play in the intestinal microecological environment, which results in sheep exhibiting different fecal scoring grades.

The core microorganism Desulfobacterota at the portal level is enriched in group F4, Desulfobacterota has also been detected in the human colon and is the dominant flora, and increased abundance of Desulfobacterota has been found to be associated with intestinal inflammation (Wang et al., 2022). *Desulfovibrio* is a sulfate-reducing anaerobic bacterium, and it has been Studies have reported that *Desulfovibrio* spp. are widespread in the gut and their increased abundance has been associated with colonic disease (Hagiya et al., 2018; Sayavedra et al., 2021). Previous studies have shown that *Peptostreptococcaceae* promote lesions in the gastric mucosa by modulating cellular inflammatory factors (Chen et al., 2019). In this study, we found that Cellvibrionales, *Cellvibrio_sp__OA_2007* were significantly enriched in group F4, while *Cellvibrionaceae*, *Cellvibrio* were significantly enriched in group F1, and that this type of genera was negatively correlated with the growth performance, which may be the reason leading to the growth performance in group F3. This may be the reason for the best growth performance of F3 group. In summary in the colon Actinobacteria, *Peptostreptococcaceae* were the key flora influencing fecal scoring and immunity. Cellvibrionales flora, *Caproiciproducens-s_uncultured_bacterium*, and *Caproiciproducens* were the key flora influencing growth performance. Acidaminococcales, and *Acidaminococcaceae* were identified to carry antibiotic resistance genes, and their relative abundance in the feces of the healthy group was significantly higher than that of the diarrhea group, which is in agreement with our findings (Díaz-Regañón et al., 2023). Gammaproteobacteria include Enterobacteriales, *Pseudomonas*, etc., which are the hallmark flora of intestinal inflammation and diarrhea (Williams et al., 2010). *Pseudoxanthomonas*, a Gram-negative bacterium, is one of the markers of pancreatic tumors and its abundance affects blood physiological and biochemical indices (Riquelme et al., 2019). In this study, *Pseudoxanthomonas* was significantly correlated with blood physiological and biochemical indices in Hu sheep. Gammaproteobacteria was positively correlated with body weight, daily weight gain, carcass length, and negatively correlated with TP and EO. Along with the accompanying maturation of the microflora, fecal microorganism have a tremendous capacity for self-regulation, and some bacteria that regulate inflammation were enriched in the high fecal score group. In summary, Acidaminococcales flora, Gammaproteobacteria, and Proteobacteria are the key genera influencing fecal scores. *Muribaculaceae*, *Oscillospiraceae-g__uncultured* are key flora affecting growth performance and immunity.

## Conclusion

In summary, based on large-scale fecal morphology observation and moisture content determination, the present study developed fecal grade scoring standards for fattening Hu sheep and found that the production performance with fecal scores between grades 3 and 4 were better than those of other grades. The composition of rumen, colon, and rectum microorganism were different in different fecal scoring groups, and the lower the fecal scoring grade, the more stable the microbial community structure. Differential microorganisms were closely related to the fecal score and growth performance of Hu sheep. The Actinobacteria, *Peptostreptococcaceae*, Acidaminococcales, Gammaproteobacteria, and Proteobacteria were closely related to fecal scoring grades, and the Cellvibrionales, *Caproiciproducens-s_uncultured_bacterium*, and *Caproiciproducens* were strongly associated with growth performance. *Muribaculaceae*, *Oscillospiraceae-g__uncultured* were strongly associated with growth performance and immunity.

## Data availability statement

The datasets presented in this study can be found in online repositories. The names of the repository/repositories and accession number(s) can be found in the article/Supplementary material.

## Ethics statement

The animal studies were approved by Institutional Animal Care and Ethics Committee of Gansu Agricultural University. The studies were conducted in accordance with the local legislation and institutional requirements. Written informed consent was obtained from the owners for the participation of their animals in this study.

## Author contributions

XY: Conceptualization, Formal analysis, Methodology, Writing – original draft. JW: Writing - review and editing. JC: Data Curation, Methodology, Writing - review and editing. DZ: Supervision, Writing - review and editing. KH: Writing - review and editing. YukZ: Data curation, Writing - review and editing. XL: Supervision, Writing - review and editing. YuZ: Supervision, Writing - review and editing. LZ: Writing - review and editing. DX: Methodology, Writing - review and editing. ZM: Writing - review and editing. JL: Writing - review and editing. ZH: Writing - review and editing. CL: Methodology, Writing - review and editing. HT: Writing - review and editing. XW: Project administration, Supervision, Writing - review and editing. WW: Resources, Supervision, Writing - review and editing. XZ: Methodology, Resources, Writing - review and editing.

## Footnotes

^1^ http://ccb.jhu.edu/software/FLASH/

^2^ http://www.drive5.com/usearch/manual/uchime_algo.html

^3^ http://huttenhower.sph.harvard.edu/galaxy/
