# Supplementary material for: Relationship between sheep feces scores and gastrointestinal microorganisms and their effects on growth traits and blood indicators
Source: Front Microbiol. 2024 Feb 14;15:1348873. doi: 10.3389/fmicb.2024.1348873 (PMC10899443; doi:10.3389/fmicb.2024.1348873)
Supplement: Supplementary file 1 [file Data_Sheet_1.DOCX]

Supplementary Material

# 1. Supplementary Figures and Tables

**1.1 Supplementary Tables**

**Supplementary Table S1.** Feed Formulation and Nutrient Levels.

| Ingredients | Percentage, % | Chemical composition | Content |
| --- | --- | --- | --- |
| Corn | 32.50 | Dry matter (DM), % | 88.78 |
| Corn germ meal | 18.00 | Crude protein (CP), % | 13.09 |
| Corn stalks | 12.00 | Digestible energy, MJ/kg | 11.11 |
| Corn hulls | 11.00 | Crude fiber (CF), % | 9.78 |
| Corn cob | 8.00 | Ether extract (EE), % | 1.72 |
| Soybean meal | 4.00 | Acid Detergent Lignin (ADL), % | 21.15 |
| Cotton meal | 5.00 | Nitrogen free extract (NFE), % | 55.05 |
| Molasses | 3.30 | Neutral detergent fiber (NDF), % | 27.08 |
| Bentonite | 1.50 | Acid detergent fiber (ADF), % | 13.99 |
| Baking soda | 1.00 |  |  |
| Stone powder | 0.80 |  |  |
| Expanded Urea | 0.70 |  |  |
| NaCl | 0.70 |  |  |
| Gypsum powder | 1.00 |  |  |
| Premix | 0.50 |  |  |
| Total | 100.00 |  |  |

*Note*: The premix included the following per kg of the starter diet: 25 mg Fe as FeSO_4_·H_2_O; 40 mg Zn as ZnSO_4_·H_2_O; 8 mg Cu as CuSO_4_·5H_2_O; 40 mg Mn as MnSO_4_·H_2_O; 0.3 mg I as KI; 0.2 mg Se as Na_2_SeO_3_; 0.1 mg Co as CoCl_2_; 940 IU vitamin A; 111 IU vitamin D; 20 IU vitamin E; and 0.02 mg vitamin B_12_.

**Supplementary Table S2**. Alpha diversity index in rumen, colon, and rectum of Hu sheep in different fecal scores groups.

| Items | Rumen | | | | Colon | | | | Rectum | | | |
| --- | --- | --- | --- | --- | --- | --- | --- | --- | --- | --- | --- | --- |
|  | Group | | | | | | | | | | | |
|  | F1 | F2 | F3 | F4 | F1 | F2 | F3 | F4 | F1 | F2 | F3 | F4 |
| No. | 128 | 239 | 112 | 8 | 11 | 9 | 12 | 4 | 15 | 10 | 14 | 6 |
| Shannon | 5.05±0.05 | 5.07±0.03 | 4.97±0.07 | 5.06±0.257 | 5.81±0.09 | 5.92±0.19 | 5.62±0.16 | 5.79±0.15 | 5.93±0.05 | 5.86±0.07 | 5.83±0.12 | 6.09±0.17 |
| Simpson | 0.97±0.00^a^ | 0.96±0.00^ab^ | 0.94±0.00^b^ | 0.95±0.027^ab^ | 0.99±0.00 | 0.99±0.01 | 0.98±0.00 | 0.99±0.01 | 0.99±0.00 | 0.99±0.00 | 0.99±0.00 | 0.99±0.00 |
| Chao1 | 801.93±15.87^b^ | 845.29±12.53^ab^ | 838.10±19.41^ab^ | 935.75±121.722^a^ | 994.54±45.56 | 1169.88±82.65 | 974.42±91.87 | 1024±86.21 | 1002.33±50.70 | 917.5±49.74 | 1076.64±75.57 | 1122±108.87 |

*Note*: Data in the table are mean ± standard error, different lowercase letters in the same row of shoulder labels indicate significant differences, the same below.

**Supplementary Table S3**. Comparative analysis of tissues and organs of Hu sheep with different fecal scores.

| Traits | | Group | | | | *P*-value |
| --- | --- | --- | --- | --- | --- | --- |
|  |  | F1 | F2 | F3 | F4 |  |
| No. | | 129 | 240 | 113 | 8 |  |
| Head | Absolute weight,kg | 2.17±0.01 | 2.22±0.02 | 2.28±0.02 | 2.21±0.09 | 0.075 |
|  | Relative weight, % | 0.045±0.00 | 0.05±0.00 | 0.05±0.00 | 0.05±0.00 | 0.649 |
| Hoof | Absolute weight,kg | 1.00±0.01^b^ | 1.02±0.01^b^ | 1.07±0.01^a^ | 0.78±0.17^c^ | 0.000 |
|  | Relative weight,% | 0.02±0.00^a^ | 0.02±0.00^a^ | 0.02±0.00^a^ | 0.02±0.00^b^ | 0.000 |
| Fur | Absolute weight,kg | 4.08±0.05^b^ | 4.09±0.04^b^ | 4.29±0.05^a^ | 3.93±0.31^ab^ | 0.015 |
|  | Relative weight,% | 0.08±0.00 | 0.08±0.00 | 0.08±0.00 | 0.08±0.00 | 0.773 |
| Heart | Absolute weight,kg | 0.18±0.00 | 0.18±0.00 | 0.19±0.00 | 0.19±0.00 | 0.068 |
|  | Relative weight,% | 0.00±0.00 | 0.00±0.00 | 0.00±0.00 | 0.00±0.00 | 0.203 |
| Liver | Absolute weight,kg | 0.81±0.01^b^ | 0.83±0.00^b^ | 0.87±0.01^a^ | 0.85±0.05^ab^ | 0.002 |
|  | Relative weight,% | 0.01±0.00 | 0.01±0.00 | 0.01±0.00 | 0.01±0.00 | 0.238 |
| Spleen | Absolute weight,kg | 0.05±0.00 | 0.05±0.00 | 0.05±0.00 | 0.06±0.00 | 0.648 |
|  | Relative weight,% | 0.00±0.00 | 0.00±0.00 | 0.00±0.00 | 0.00±0.00 | 0.529 |
| Lung | Absolute weight,kg | 0.59±0.00^b^ | 0.60±0.00^b^ | 0.63±0.01^a^ | 0.61±0.02^ab^ | 0.017 |
|  | Relative weight,% | 0.01±0.00 | 0.01±0.00 | 0.01±0.00 | 0.01±0.00 | 0.473 |
| Kidney | Absolute weight,kg | 0.12±0.00^b^ | 0.12±0.00^b^ | 0.13±0.00^a^ | 0.12±0.00^ab^ | 0.027 |
|  | Relative weight,% | 0.00±0.00 | 0.00±0.00 | 0.00±0.00 | 0.00±0.00 | 0.886 |

**Supplementary Table S4**. Volatile fatty acids in the rumen of Hu sheep in different fecal scores groups.

| Group | F1 | | F2 | | F3 | | F4 | |
| --- | --- | --- | --- | --- | --- | --- | --- | --- |
| No. | 123 | | 229 | | 106 | | 8 | |
| Items | mean | se | mean | se | mean | se | mean | se |
| Acetic acid | 39.17 | 2.40 | 37.77 | 1.89 | 38.95 | 2.01 | 42.12 | 10.59 |
| Propionic acid | 14.82 | 0.64 | 14.29 | 0.59 | 14.71 | 0.65 | 15.56 | 2.38 |
| Isobutyric acid | 1.34 | 0.08 | 1.44 | 0.10 | 1.39 | 0.09 | 1.95 | 0.72 |
| Butyrate | 6.95 | 0.27 | 6.72 | 0.25 | 7.11 | 0.31 | 8.44 | 2.01 |
| Isovaleric acid | 1.40 | 0.06 | 1.48 | 0.07 | 1.47 | 0.07 | 1.70 | 0.35 |
| Valeric acid | 0.80^b^ | 0.06 | 0.69^b^ | 0.03 | 0.72^b^ | 0.04 | 1.23^a^ | 0.53 |
| Total acid | 67.35 | 3.28 | 65.25 | 2.77 | 67.24 | 2.93 | 73.87 | 15.85 |
| Acetic acid% | 0.57 | 0.01 | 0.57 | 0.00 | 0.56 | 0.01 | 0.54 | 0.02 |
| Propionic acid% | 0.22 | 0.00 | 0.22 | 0.00 | 0.22 | 0.00 | 0.23 | 0.02 |
| Isobutyric acid% | 0.02 | 0.00 | 0.02 | 0.00 | 0.02 | 0.00 | 0.02 | 0.00 |
| Butyrate% | 0.11 | 0.00 | 0.11 | 0.00 | 0.11 | 0.00 | 0.11 | 0.01 |
| Isovaleric acid% | 0.02 | 0.00 | 0.02 | 0.00 | 0.02 | 0.00 | 0.02 | 0.00 |
| Valeric acid% | 0.01^ab^ | 0.00 | 0.01^b^ | 0.00 | 0.01^b^ | 0.00 | 0.01^a^ | 0.00 |

**Supplementary Table S5**. Comparison of mutton quality of Hu sheep in different fecal scores groups.

| Group | F1 | | F2 | | F3 | | F4 | |
| --- | --- | --- | --- | --- | --- | --- | --- | --- |
| No. | 123 | | 232 | | 111 | | 8 | |
| Items | mean | se | mean | se | mean | se | mean | se |
| Fat | 4.05 | 0.14 | 4.03 | 0.09 | 3.87 | 0.12 | 3.55 | 0.29 |
| Water | 71.93^b^ | 0.12 | 71.97^b^ | 0.09 | 72.21^b^ | 0.14 | 73.60^a^ | 0.46 |
| Salt | 1.88 | 0.05 | 1.85 | 0.04 | 1.79 | 0.05 | 1.98 | 0.21 |
| Protein | 22.05^a^ | 0.13 | 22.18^a^ | 0.10 | 22.39^a^ | 0.12 | 20.82^b^ | 0.66 |
| Collagen | 1.28^a^ | 0.03 | 1.31^a^ | 0.02 | 1.26^a^ | 0.03 | 1.06^b^ |  |

## 1.2 Supplementary Figures


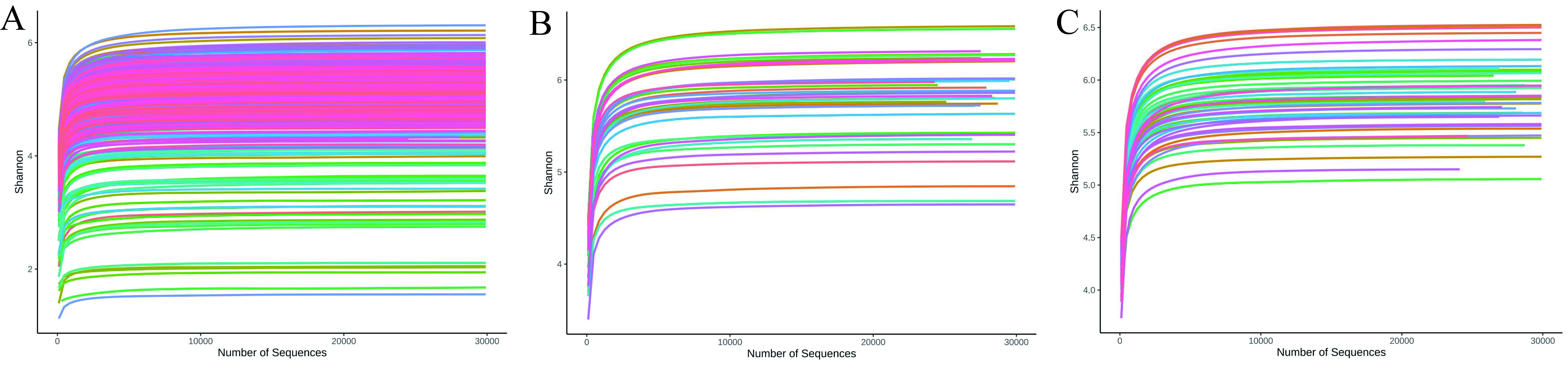
**Supplementary** **Figure S1.** Dilution curves. (A. rumen, B. colon; C. rectum).

**Supplementary Figure S2.**
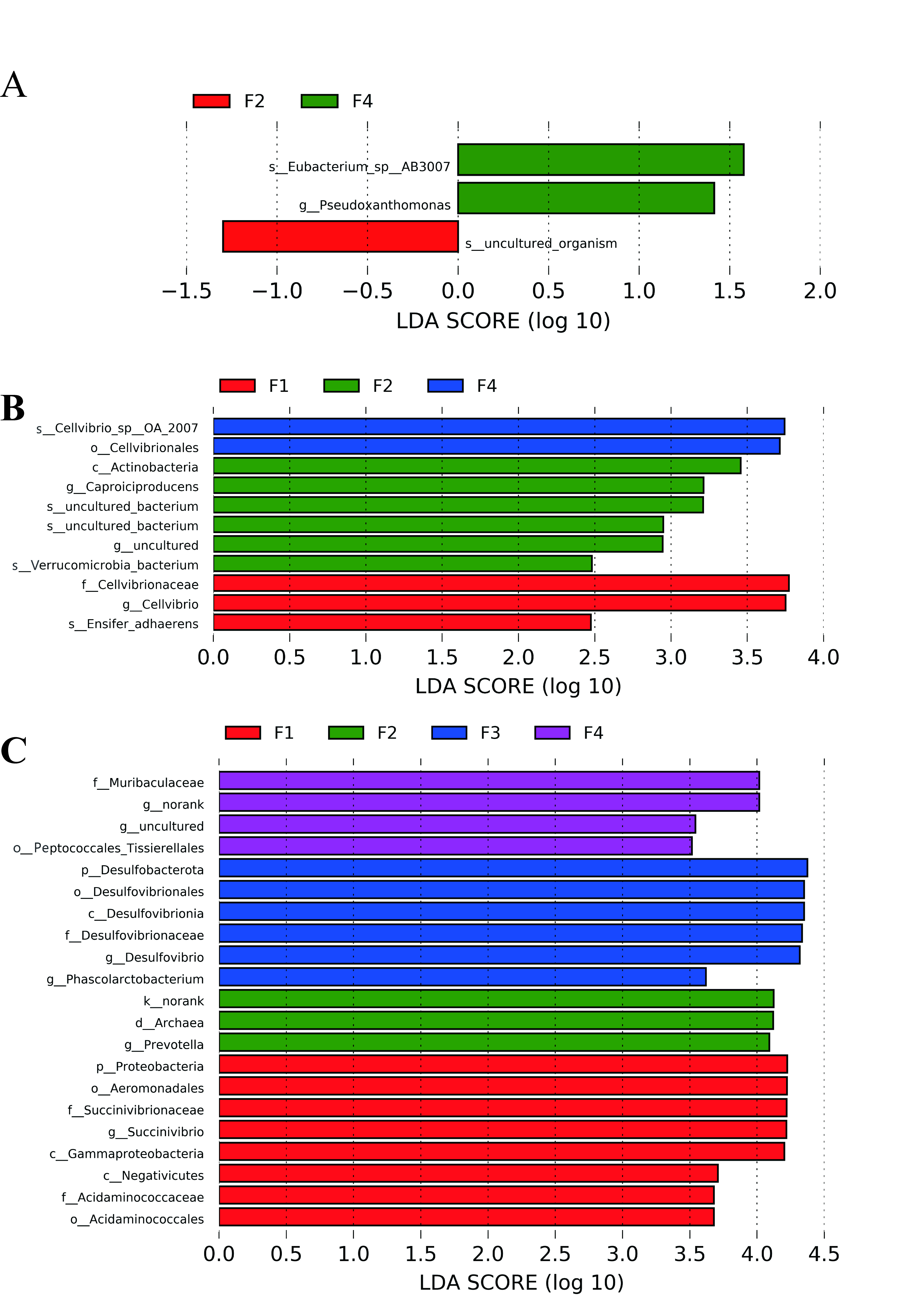
 LEfSe analysis of Hu sheep with different fecal scores. A. rumen; B. colon; C. rectum.
